# The Use of Geostatistics in the Study of Floral Phenology of *Vulpia geniculata* (L.) Link

**DOI:** 10.1100/2012/624247

**Published:** 2012-05-01

**Authors:** Eduardo J. León Ruiz, Herminia García Mozo, Eugenio Domínguez Vilches, Carmen Galán

**Affiliations:** Department of Botany, Ecology and Plant Physiology, University of Córdoba, 14071, Córdoba, Spain

## Abstract

Traditionally phenology studies have been focused on changes through time, but there exist many instances in ecological research where it is necessary to interpolate among spatially stratified samples. The combined use of Geographical Information Systems (GIS) and Geostatistics can be an essential tool for spatial analysis in phenological studies. Geostatistics are a family of statistics that describe correlations through space/time and they can be used for both quantifying spatial correlation and interpolating unsampled points. In the present work, estimations based upon Geostatistics and GIS mapping have enabled the construction of spatial models that reflect phenological evolution of *Vulpia geniculata* (L.) Link throughout the study area during sampling season. Ten sampling points, scattered troughout the city and low mountains in the “Sierra de Córdoba” were chosen to carry out the weekly phenological monitoring during flowering season. The phenological data were interpolated by applying the traditional geostatitical method of Kriging, which was used to ellaborate weekly estimations of *V. geniculata* phenology in unsampled areas. Finally, the application of Geostatistics and GIS to create phenological maps could be an essential complement in pollen aerobiological studies, given the increased interest in obtaining automatic aerobiological forecasting maps.

## 1. Introduction

Phenology has been defined as “the study of the timing of recurrent events, the causes of their timing with regard to biotic and abiotic forces, and the interrelation among phases of the same or different species” [1]. Because phenology is genetically conditioned but also controlled by environmental factors, recent phenological studies have acquired a new dimension and scientific significance, as they provide direct information to know how species are being affected by global change [2–5].

Poaceae family comprises more than 700 genera with about 10.000 species [6]. This family includes both annual and perennial herbs, which are essentially anemophilous. In most cases grasses present a high number of flowers per inflorescence that release a high quantity of pollen grains to the atmosphere. Many species are well distributed into and around cities, which, besides the high allergenicity of the grass pollen grain, make these species the main cause of pollinosis [7]. The present study is focused on the phenology of *Vulpia geniculata* (L.) Link, one of the most common grass species in the city and low mountains of the “Sierra de Córdoba” (Southwestern Spain). *V. geniculata* is also one of the 4 species that produce more pollen per inflorescence within the study area, as a previous study revealed [8].

Georeferenced data, such as floral phenology of a population, can be incorporated into a GIS to produce map layers. While the advent of GIS allows for compiling and manipulating spatially referenced data, modelling spatial patterns from areas where no data are available is difficult without an adequate set of statistical tools [9]. GIS are computed-based methodologies conceived for spatial data collection, storage, retrieval, transformation, display, and analysis [10]. Geostatistics designs a group of tools and techniques that are useful to analyze spatial patterns and predict the values of a continuous variable distributed in space or in time at unsampled points [11]. 

The combined use of GISs and geostatistics has been demonstrated as a very valuable method for spatial analysis in environmental studies and also plant distribution [12–14]. Both tools applied on floral phenology studies will contribute to create phenological maps in base of a limited number of sampled locations [15]. In the present study geographic Information Systems (GISs) and geostatistics tools have been applied to analyse the temporal phenological evolution and spatial distribution of *V. geniculata* (L.) Link in the city of Cordoba and in the Sierra of Cordoba.

The study of floral phenology dynamic in groups of species with stenopalynous pollen, such as grasses, allow us a better understanding of the aerobiological curves [16]. The aim of this work is to study the spatial variations in floral phenology of *V. geniculata* during the spring of the years 2004–2006, and also, to create phenological maps throughout the study area, starting out of a limited number of sampling points. We also have tried to compare the obtained maps with Poaceae pollen emission in the atmosphere in order to demonstrate which areas more contribute to the pollen curve.

## 2. Material and Methods

### 2.1. Study Area

The study was carried out in the outskirts of the city of Córdoba 37°50′N and 4°45′W; 123 m a.s.l., South-western Spain. The climate is Mediterranean with some continental features, and it is characterised by temperate, cold winters and dry, cold summers, with an annual average temperature of 17.6°C and an annual rainfall 536 mm, average data from 1971 to 2000 [17].

### 2.2. Aerobiological Sampling

Aerobiological data have been obtained following the Spanish Aerobiology Network Management and Quality Control [18]. The aerobiological sampler used was a Hirst-type spore trap located on the roof of the Faculty of Education, University of Cordoba.

### 2.3. Phenological Survey

**Figure 1:**
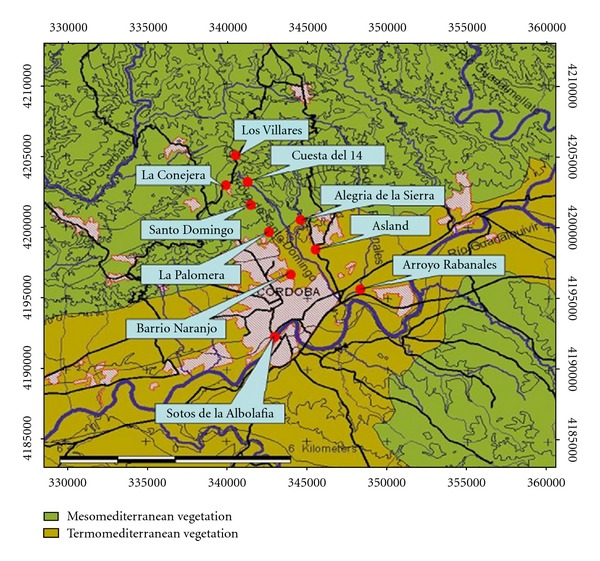
Sampling points distribution.

The phenological monitoring took place during years 2004–2006. Ten sampling points were randomly chosen into the study area (Figure 1). Five of them belonged to the Termomediterranean bioclimatic zone, and five belonged to the Mesomediterranean [19].  Table 1 shows coordinates, altitude and bioclimatic zone for each one of the chosen points.

The flowering period has been divided into five fenophases for the study, defined according to the number of open flowers in the inflorescence, based upon the methodology proposed by [20]. It has been considered open flowers those in which it has been observed exerted stamens. The phase before flowering, or phase 0, comprises a period of time that begins with emergence of the inflorescence and finishes when the first blooming occurs. The start flowering phase, or phase 1, lasts until the opening of approximately the 25% of the flowers. The full flowering phase, or phase 2, comprises the period of maximum pollen shedding and lasts until the opening of approximately the 75% of the flowers. The ending flowering phase, or phase 3, comprises the opening of the last 25% of the flowers and finishes when all the anthers have released the majority amount of pollen. The past flowering phase, or phase 4, begins when all the anthers are almost empty.

Phenological observations were carried out once a week from 2004 to 2006. In order to calculate an average phenological value per area, a quadrant measuring 1 m^2^ was randomly deposited five times in each area. 5-mean phenological value was assigned at each sampling point. 

### 2.4. Geostatistical Analysis

The geostatistical analysis was performed by using the software geostatistics for Environmental Sciences (GS+). Variables used in geostatistical analysis were the floral phenological data recorded for the dates when *Vulpia geniculata* individuals were present in every sampling point and the geographical coordinates.

The steps followed for the geostatistical analysis were performed following the proposed methodology of Moral [21].

#### 2.4.1. Descriptive Statistical Analysis

1. Univariant statistical analysis: mean and standard deviation, maximum and minimum values, and variation coefficient were calculated for the different date phenological datasets.
2. Data exploratory analysis to detect the presence of outliers. This analysis consisted in the calculation of a lower and upper threshold, below or above which any value is considered an outlier. This threshold is equivalent to
  (1) m±3s,
  where m is the mean value of each dataset an s is the standard deviation.

#### 2.4.2. Structural Analysis

Variance characteristics by means of a variogram analysis were studied. First of all, a theoretical variogram was chosen. In our case, the phenological variable is a very continuous spatial phenomenom, so we decided to use gaussian variograms to study each phenological dataset. Then, these variograms should be adjusted to the data we have. As Moral [21] remarks, variogram modelling should be performed by the user, in accordance to the knowledge of the spatial behaviour of the variable. When modelling we tried to obtain *r* ^2^ coefficients close to 1, and low RSS values.

#### 2.4.3. Validation and Interpolation

Cross-validation analyses were performed to estimate the goodness of the chosen variograms. In these analyses each measured point in a spatial domain is individually removed from the domain and its value estimated via kriging as though it were never there:

(2) $$\begin{array}{c} Z^{*}\left(v\right)=\sum \lambda_{i}Z\left(x_{i}\right)+m\left(1-\sum \lambda_{i}\right), \end{array}$$

Z*(*v*) is estimated value, *Z*(*x*_*i*_)  is  1,…, *n* sampling values,  *λ*_*i*_ is Linear estimation constant which reduces the variance up to zero, *m*  is  *x*_(*i*+1)/2_ if *i* is an odd number, and *m* = *x*_(*i*/2)_ + (*x*)_(*i*/3)_ if *i* is an even number.

As a result of these cross-validation analysis different regressions comparing estimated versus actual values were obtained for each sampling date. The obtained regression coefficients show the goodness of the interpolation, so the closer they are to 1 the better the regression is and the more the function fits the data. Finally, we proceed to interpolate the values for unsampled points in the study area. In this paper we decided to use the Simple Kriging [22, 23], in which interpolation estimates are made based on values at neighbouring locations in addition to the knowledge about the underlying spatial relationships in a data set calculated by variograms. These interpolation values were used to obtain phenological maps of the area during survey season. We used the software ArcView 3.2 to obtain these maps. In order to compare these values with the poaceae pollen concentrations, we considered that an area showing a value between 1.5 and 2.5 corresponds to populations of *Vulpia geniculata* that are liberating pollen at its maximum (full flowering period), and so we can find out which areas contribute the most to pollen curve.

## 3. Results

### 3.1. Aerobiological Survey

**Figure 2:**
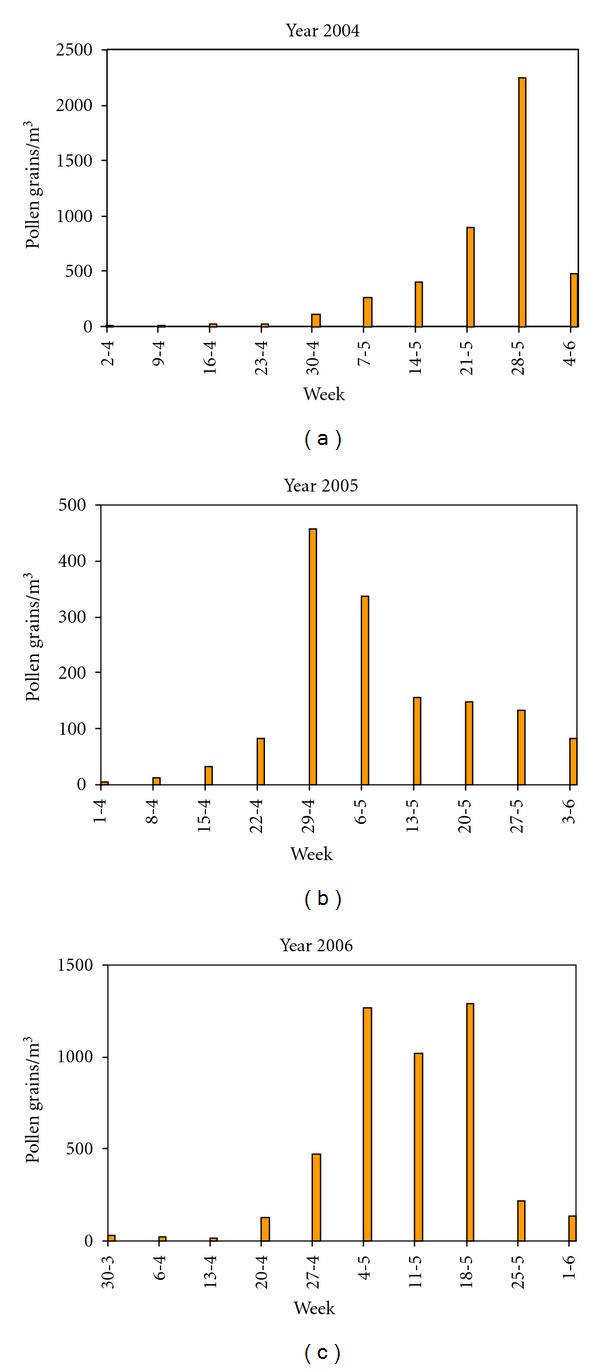
Poaceae airborne pollen cumulated per week during the study years.

Using the data obtained in the aerobiological sampling, graphs showing pollen index accumulated per week have been elaborated for each year of study (Figure 2). The highest pollen concentration was recorded during year 2004 (Figure 2(a)), while the lowest was registered during year 2005 (Figure 2(b)). We also noticed that in year 2004 highest pollen concentration was recorded during last week of May, while in 2005 it occurred during last week of April. Both phenomena could be explained by the precipitations registered in spring for the three years of study, since during year 2004 abundant precipitations were registered in April and May, while in year 2005 rainfall was scarce during those months, being this year one of the driest in the last 4 decades. In year 2006 intermediate pollen counts were obtained related to normal climatic characteristics of this year (Figure 2(c)).

### 3.2. Geostatistical Analysis

Results of descriptive statistical analysis are shown in Tables 2, 3 and 4. It is observed that Variance is not significant among the different survey seasons. Results also indicated that no outliers were present in any of the datasets.

**Figure 3:**
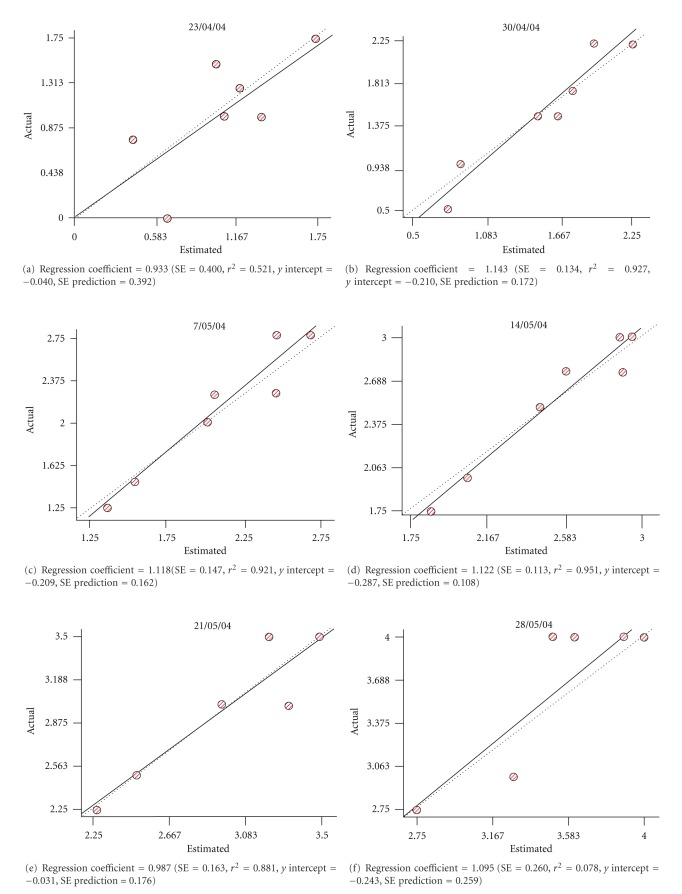
Cross-validation analyses (variograms) obtained for year 2004.

**Figure 4:**
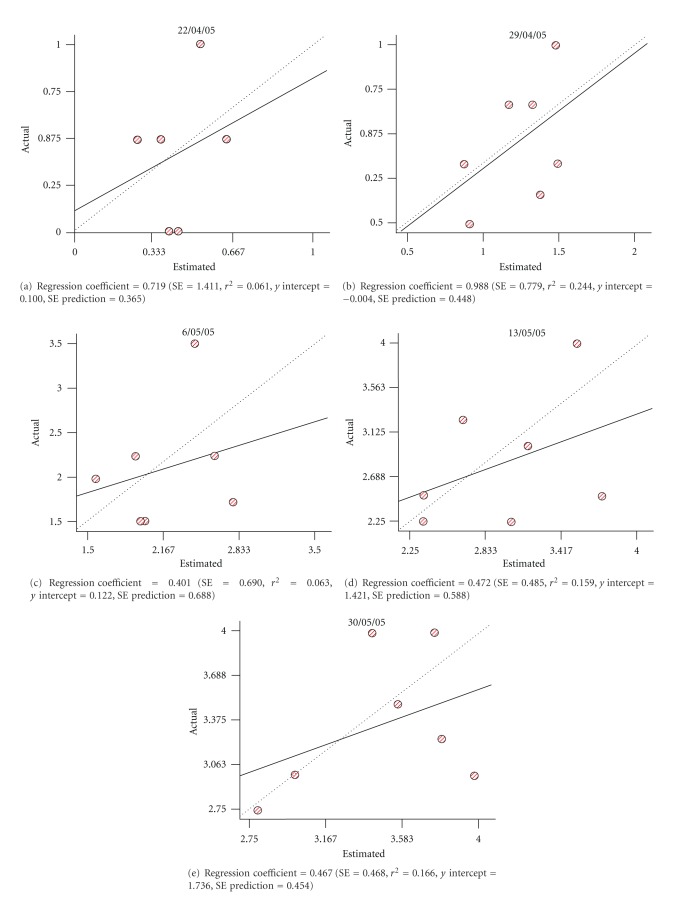
Cross-validation analyses (variograms) obtained for year 2005.

**Figure 5:**
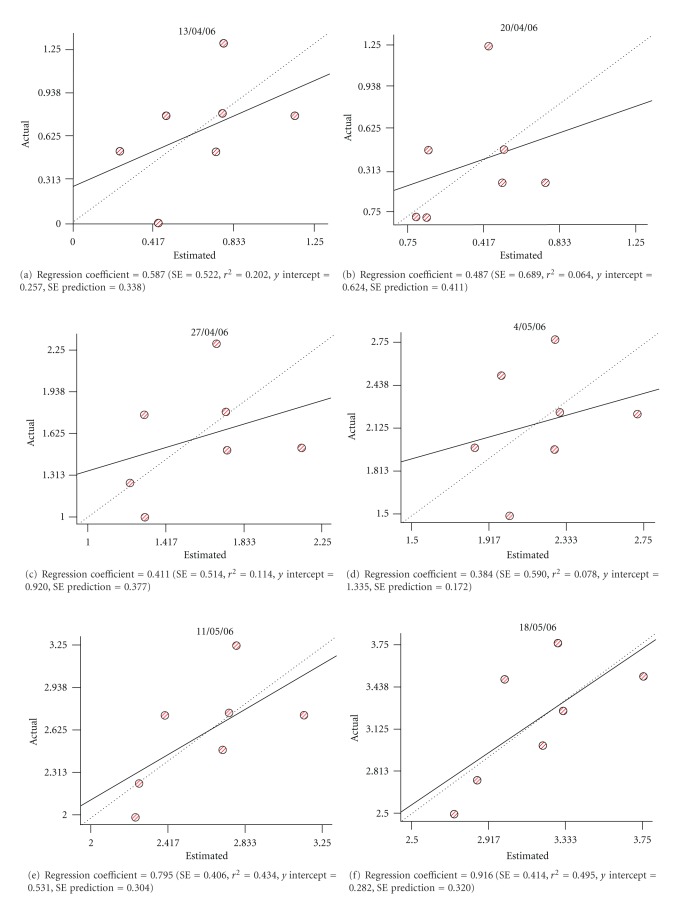

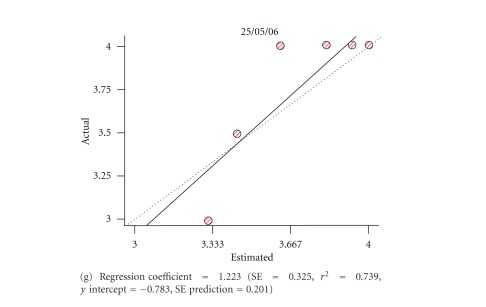
Cross-validation analyses (variograms) obtained for year 2006.

Regarding to the structural analysis the variograms obtained for each dataset are shown in Figures 3, 4 and 5. In all the analysed dates, it was possible to adjust a gaussian isotropic model to study spatial variance of phenological variable. Best results were obtained in year 2006 (Figure 5), due to RSS and *r* ^2^ values were close to 0 and 1, respectively.

Results of the cross-validation analysis are shown in Table 5. The regression coefficient represents a measure of the goodness of fit for the model describing the lineal regression equation. In general we observe that regression coefficients were close to 1, which shows the goodness of the interpolation. In the cases where those coefficients are not that close, it has to be taken into consideration when proceeding to evaluate the quality of the interpolation. 

**Figure 6:**
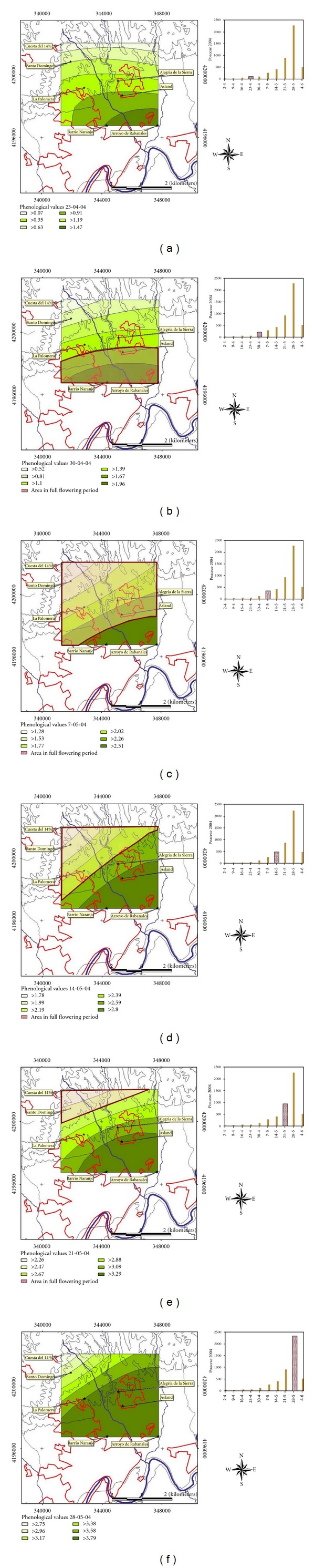
Phenological maps. Year 2004.

**Figure 7:**
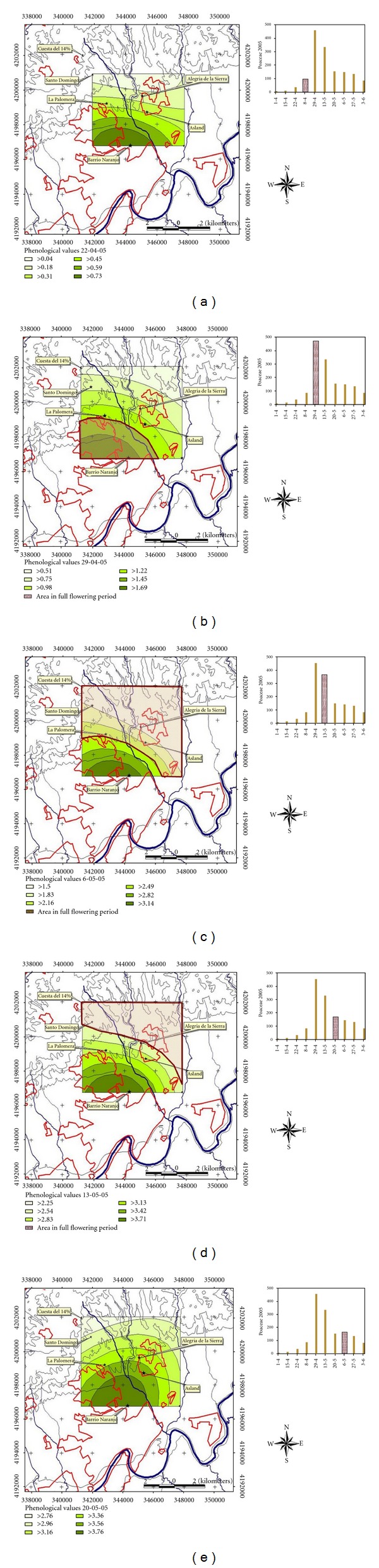
Phenological maps. Year 2005.

**Figure 8:**
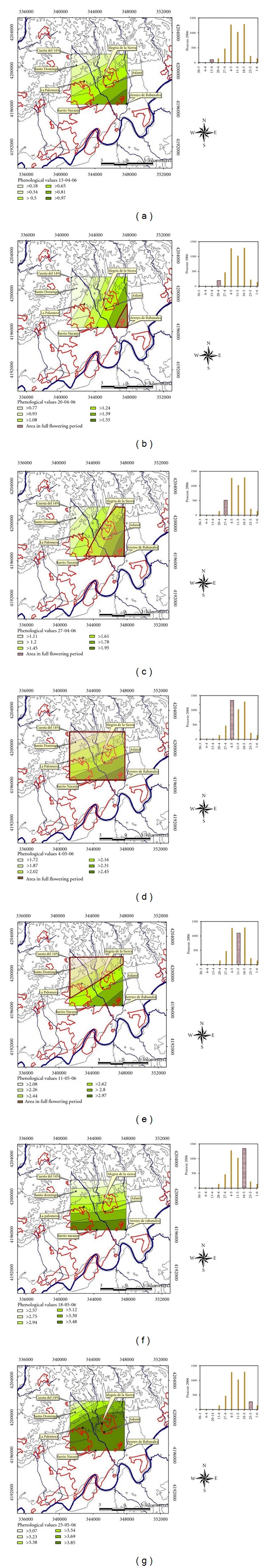
Phenological maps. Year 2006.

All the maps obtained are shown in Figures 6, 7 and 8. In general, it is observed that the zone where flowering phenology is more advanced is placed in the southern part of the map, which coincides with the lowest elevation in the study area. Otherwise, the zone where the flowering phenology is more delayed is always placed in the northern top of the map. During years 2004 and 2006 the maps show that populations placed on the Northwest, at higher altitudes, contribute the most to the higher poaceae pollen concentration in the air (Figures 6 and 8). On the contrary, during year 2005, populations placed on the south, at lower altitudes, contribute the most to the hightest poaceae pollen concentration in the air, recorded during the week from April 29th to May 05th (Figure 7). Nonetheless, populations placed in the north area seem to contribute to high concentrations registered during subsequent weeks, as well.

## 4. Discussion

Most phenomena show, as an inherent feature, a high degree of spatial continuity Moral [21]. In ecological research, there are many instances where it is necessary to interpolate among spatially stratified samples which is the reason because in last years GISs and geostatistics are being applied to environmental studies such as entomology [9] plant distribution [13, 24], and general ecology [25] with excellent results.

Traditionally plant flowering phenological studies have been focused on changes through time. Patterns across space remain largely unexplored. Only few works have developed models in terms of both space and time [26–28]. 

In the present paper the working hypothesis that starting from several sampling points randomly distributed through the city and the Sierra Norte of Córdoba we would be able to obtain valuable phenological information of *V. geniculata* was confirmed. By using georeferenced data, phenological maps of a given area can be constructed. Kriged estimates were subsequently used to map phenological variations to visualize changes through the space. The present work indicates that *V.geniculata* phenology is highly associated to altitude and topographic situation.

In recent years, there has been a surge of interest in phenology as an indicator of global climate change effects, particularly during spring months [29, 30]. In this context, located plant phenology data and phenological maps provide real data as how the climate affects the phenology of plants and their possible adaptations to new climate scenarios [5, 31]. Systematic future measurement of floral phenology in located points could provide extended maps that would offer valuable information about the biological impacts of climate change on different species.

In relation to the use of validated phenological maps constructed by interpolation we can conclude that the obtained maps are a valuable tool to interpret grass airborne pollen concentrations registered in Cordoba. This work shows that the populations located at higher altitude seem to contribute more to the Poaceae pollen curve. Nevertheless, in those years when the main pollen season occurs earlier, due to specific meteorological conditions, those populations placed at lower altitude, which start their flowering in advance, also seem to play an important role. So, given that grasses surrounding urban areas are a huge source of pollen, the knowledge of the distribution and evolution of flower phenology from a limited number of sampling points will provide high value data in bio-pollutant environmental studies. Future applications of these tools may extend to the spatial analysis of airborne pollen data especially on the location of main pollen emission and airborne spatial dispersion.

## Figures and Tables

**Table 1:** Sampling points coordinates.

Sampling point	Altitude	*X* coordinate	*Y* coordinate	Bioclimate
Sotos de la Albolafia	92	343274	4193395	Termomediterranean
Arroyo de Rabanales	109	357483	4196492	Termomediterranean
Barrio Naranjo	146	344300	4196752	Termomediterranean
Asland	184	345252	4198511	Termomediterranean
Algería de la Sierra	217	344926	4199407	Termomediterranean
La Palomera	251	342737	4198985	Mesomediterranean
Santo Domingo	368	341758	4200722	Mesomediterranean
Cuesta del 14%	511	341181	4201771	Mesomediterranean
La Conejera	555	340584	4201852	Mesomediterranean
Los Villares	585	341149	4203129	Mesomediterranean

**Table 2:** Results of Descriptive Analysis. Year 2004.

2004	23 Apr	30 Apr	07 May	14 May	21 May	28 May
Mean	1.04	1.54	2.11	2.54	3.04	3.68
Variance	0.32	0.40	0.33	0.24	0.26	0.31
Minimum	0.00	0.50	1.25	1.75	2.25	2.75
Maximum	1.75	2.25	2.75	3.00	3.50	4.00
*n*	7	7	7	7	7	7
Upper threshold	2.74	3.44	3.83	4.00	4.56	5.34
Lower threshold	−0.67	−0.37	0.38	1.07	1.51	2.02

**Table 3:** Results of Descriptive Analysis. Year 2005.

2005	22 Apr	29 Apr	06 May	13 May	20 May
Mean	0.42	1.18	2.11	2.82	3.25
Variance	0.14	0.26	0.48	0.41	0.20
Minimum	0.00	0.50	1.50	2.25	2.75
Maximum	1.00	2.00	3.50	4.00	4.00
*n*	6	7	7	7	7
Upper threshold	1.55	2.72	4.18	4.74	4.59
Lower treshold	−0.71	−0.37	0.04	0.90	1.91

**Table 4:** Results of Descriptive Analysis. Year 2006.

2006	13 Apr	20 Apr	27 Apr	04 May	11 May	18 May	25 May
Mean	0.64	1.14	1.57	2.18	2.61	3.18	3.79
Variance	0.14	0.18	0.16	0.16	0.16	0.20	0.15
Minimum	0.00	0.75	1.00	1.50	2.00	2.50	3.00
Maximum	1.25	2.00	2.25	2.75	3.25	3.75	4.00
*n*	7	7	7	7	7	7	7
Upper threshold	1.78	2.43	2.77	3.38	3.82	4.53	4.97
Lower treshold	−0.49	−0.15	0.37	0.98	1.39	1.83	2.61

**Table 5:** Cross-validation summary results.

2004	23/04/04	30/04/04	07/05/04	14/05/04	21/05/04	28/05/04	
Regression coefficient	0.93	1.14	1.12	1.12	0.99	0.78	

2005	22/04/05	29/04/05	06/05/05	13/05/05	20/0505		
Regression coefficient	0.73	0.99	0.41	0.48	0.47		

2006	13/04/06	20/04/06	27/04/06	04/05/06	11/05/06	18/05/06	25/05/06
Regression coefficient	0.59	0.47	0.41	0.38	0.80	0.92	1.12

## Abbreviations

GIS: Geographic information system.
